# Ophiasis Pattern Alopecia Areata in an Infant

**DOI:** 10.7759/cureus.44920

**Published:** 2023-09-08

**Authors:** Muna Shakhashiro, Sandro Pasagic, Chase L Wilson

**Affiliations:** 1 Internal Medicine, University of Kentucky College of Medicine, Lexington, USA; 2 Dermatology, Elkhorn Dermatology, Guyana, USA

**Keywords:** infantile alopecia, cultural hair practice, ophiasis alopecia, pediatric dermatology, alopecia areata

## Abstract

Alopecia areata (AA), an autoimmune inflammatory disorder causing non-scarring hair loss, predominantly affects the adult population and is rarely encountered in young infants and neonates. The etiology of this condition remains multifactorial, involving complex interactions between genetic, autoimmune, and environmental factors. In this report, we present a notable case of a four-month-old infant who presented with distinct band-like hair loss on the right inferior lateral forehead, left inferior lateral forehead, and superior middle forehead following a culturally significant head-shaving ritual known as Chudakarana. This unique presentation of ophiasis AA in an infant is an unusual occurrence and has been associated with a poor prognosis. The patient received topical treatment with triamcinolone 0.1% lotion, resulting in improvement of alopecia at the six-week follow-up, although complete resolution of symptoms was not achieved. This case highlights the significance of recognizing atypical presentations of AA in the pediatric population and underscores the complexities in associated cultural factors.

## Introduction

Alopecia areata (AA) is an autoimmune inflammatory process that results in the sudden onset of well-circumscribed patches of non-scarring alopecia. This type of hair loss affects both men and women of various racial and ethnic backgrounds. In AA, the immune system mistakenly attacks the hair follicles; however, the etiology remains both controversial and unknown. Many researchers suspect a combination of genetic and environmental factors [1]. The appearance of AA varies widely and can present in various subtypes including patchy alopecia, ophiasis (band-like hair loss) alopecia, alopecia totalis (nearly all of the hair on the head), and alopecia universalis (total hair loss everywhere). Overall, there is approximately a 2% risk of developing AA over one’s lifetime [2]. The average age of onset is 32.2 years with a standard deviation of 14.8 years [3]. Incidence among infants and newborns is quite rare [4]. To our knowledge, fewer than 10 cases of AA presenting in infants less than six months of age have been reported [5]. 

We present a case of patchy hair loss in a four-month-old female infant weeks after undergoing a newborn shaving of the head, known as Chudakarana in Hindu tradition. The shaving of a newborn’s head is a practice prevalent in numerous cultures and is particularly common among Islamic and Hindu followers. There is no current evidence or studies that report hair loss subsequent to this ritual.

## Case presentation

A four-month-old female infant was referred to the Elkhorn Dermatology clinic for patches of alopecia on the scalp. The patient was noted by her parents to have had a full head of hair at birth. She underwent a traditional shaving of the scalp at one week of age, known as Chudakarana, and in the subsequent weeks was noticed to have areas of poor hair growth relative to surrounding scalp hair. Parents were initially concerned that the shaving practice damaged the scalp leading to alopecia.

**Figure 1:**
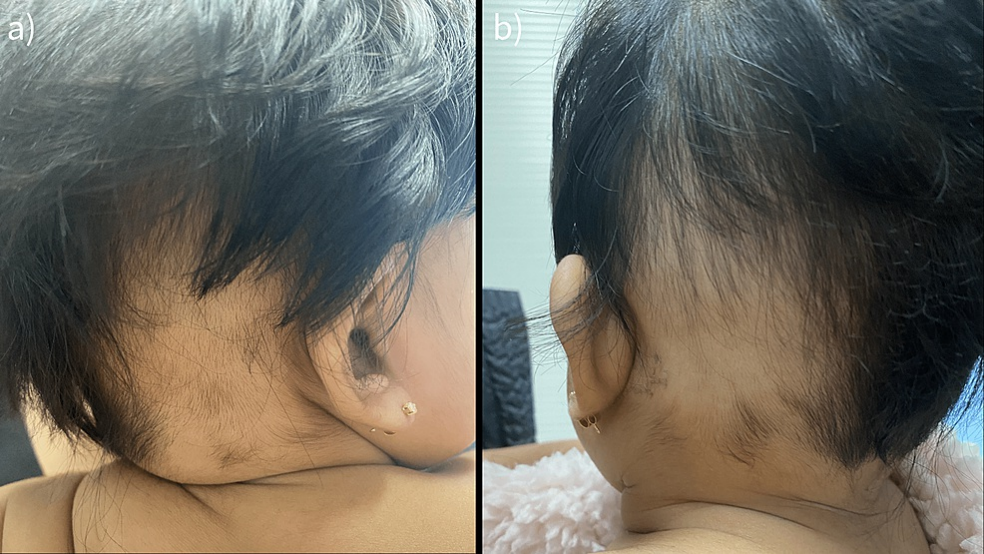
Initial visit: band-like pattern of hair loss in the (a) right and (b) left parieto-occipital areas extending toward the temples.

On examination, the child had discrete alopecic patches with preservation of the follicular ostia and terminal hairs in varying stages of regrowth distributed on the bilateral occipital scalp (ophiasis pattern) surrounded by well-formed dense terminal hair growth on the rest of the scalp (Figure 1). Nail pitting was absent and the remainder of the skin examination was unremarkable. The patient was delivered vaginally to a 31-year-old primigravida at full term with a normal course of labor. No gestational or birth complications were noted. No hair abnormalities were present at birth. Family history was significant for atopic dermatitis in her father. There was no family history of AA, skin disease, or other autoimmune conditions.

Of note, the patient’s urine CMV was positive with over 140000 IU/mL at two months of age. This was discovered after a visit to the pediatrician due to concerns of an upper respiratory infection. There was no evidence that suggested congenital infection (such as chorioretinitis, intraventricular calcifications, hearing loss, and rash).

**Figure 2:**
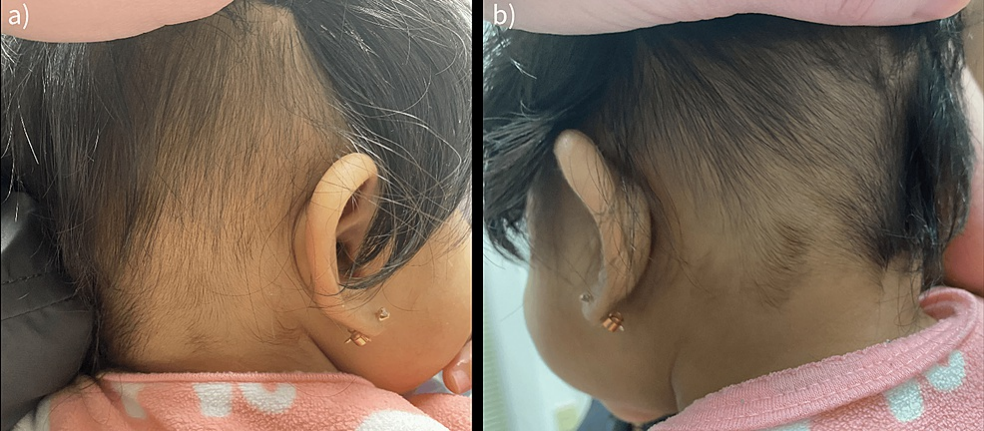
Six-week follow-up: (a) right parieto-occipital and (b) left parieto-occipital terminal hair regrowth with fairly even density and hairs in varying stages of regrowth and length.

The differential diagnosis at the time favored ophiasis pattern AA but neonatal occipital alopecia remained in our differential. Therapy was initiated with triamcinolone acetonide 0.1% lotion applied twice a day to affected areas of alopecia. At her six-week follow-up, the patient presented with additional terminal hair regrowth (Figure 2).

## Discussion

AA is an autoimmune-mediated form of nonscarring alopecia that is most often clinically diagnosed and commonly appears as well-circumscribed patches of nonscarring hair loss [6]. Confirmation with biopsy and histopathology can be of use in uncertain cases. Its prevalence has been reported at 0.1% with a lifetime incidence of approximately 2% [7]. Recent studies suggest the prevalence of AA to be greatest among pediatric populations; however, incidence among infants and newborns is considered to be quite rare with few reported cases [4,8]. The appearance of AA varies widely and can present in several patterns including patchy alopecia ophiasis, alopecia totalis, and alopecia universalis.

We report an additional rare case of AA in an infant, presenting uncommonly with the ophiasis pattern. Ophiasis pattern AA is a subtype of AA that presents as band-like hair loss in the parieto-occipital area and extends toward the temples [9]. Its prevalence among adults and children was found to be quite rare at 0.02% in a meta-analysis of the epidemiology of AA and its subtypes [2]. Ophiasis pattern AA has also reportedly been associated with a poorer prognosis [10]. Age at onset, too, is an important prognostic indicator of AA, with younger age at onset associated with greater disease severity and less favorable prognosis [10,11]. Given the poor prognosis associated with young age and subtype, AA must be differentiated from other forms of pediatric hair loss. These include neonatal occipital alopecia, friction-induced hair loss, and halo scalp ring, which is associated with trauma. The timeline of hair loss development, the lack of history of localized pressure to the occiput, and the improvement with topical corticosteroid therapy are all factors that point toward AA. Dermoscopic findings such as yellow dots, exclamation point hairs, and nail pitting are additional features that support a diagnosis of AA.

The exact pathogenesis of AA remains unknown; however, numerous genetic and environmental factors including viral infections have been proposed to play a role. There is a strong association between AA and other autoimmune diseases such as Graves disease, Hashiomoto’s thyroiditis, asthma, allergic rhinitis, psoriasis, and vitiligo [3]. Viral infections in particular have been implicated to play a pathogenic role in various autoimmune diseases. Studies have suggested cytomegalovirus and Epstein-Barr virus to be possible triggers for AA; however, this association has been refuted and evidence is not strongly supportive due to the lack of larger associated studies [12,13,14]. Although no strong correlation is present in current literature, it is intriguing to consider whether CMV infection served as a trigger for our young patient’s hair loss. Other environmental factors such as psychological stress have been implicated as a pathogenetic trigger in AA. To our knowledge, hair shaving or other physical stimuli have not been documented as a trigger of AA. The onset of alopecia following the patient’s ritualistic/traditional hair shaving is likely coincidental and unassociated with this cultural practice. 

## Conclusions

AA is a hair loss disorder that affects individuals of all ages and can present in various patterns, including the rare ophiasis pattern. This report adds to the existing literature by presenting a rare case of ophiasis AA in an infant with a history of neonatal ritual head shaving. Age of onset and subtype of AA can be important indicators of prognosis, with younger onset and ophiasis pattern associated with more severe disease. Understanding the various AA subtypes and maintaining a high index of suspicion for AA, even in atypical presentations, is crucial for early recognition and treatment to prevent progression. Our patient was treated with topical triamcinolone 0.1% lotion, resulting in an improvement of alopecia. Other factors indicative of AA include nail pitting, the presence of exclamation point hairs, and histopathological findings.

Although various environmental factors have been implicated in the pathogenesis of AA, there are no existing reports indicating that activities such as hair shaving or other physical stimuli can serve as triggers for AA. The correlation between a patient's traditional hair shaving and the onset of alopecia is likely coincidental, with no association to this cultural practice. Families can find comfort in knowing that the condition is unlikely caused by this tradition.
